# Heat shock protein family A member 8 is a prognostic marker for bladder cancer: Evidences based on experiments and machine learning

**DOI:** 10.1111/jcmm.17977

**Published:** 2023-09-28

**Authors:** Yang Liu, Zhong‐qi Pang, Jian‐she Wang, Jin‐feng Wang, Jia‐xin He, Bo Ji, Lu Zhang, Ming‐hua Ren

**Affiliations:** ^1^ Department of Urinary Surgery First Affiliated Hospital of Harbin Medical University Harbin Heilongjiang China

**Keywords:** bladder cancer, HSPA8, immune infiltration, prognostic maker, risk model

## Abstract

Heat shock protein member 8 (HSPA8) is one of the most abundant chaperones in eukaryotic cells, but its biological roles in bladder cancer (BC) are largely unclear. First, we observed that HSPA8 was abundant in both cell lines and tissues of BC, and the HSPA8‐high group had poorer T stages and overall survival (OS) than the HSPA8‐low group in the TCGA patients. Next, when we knocked down HSPA8 in BC cells, the growth and migration abilities were significantly decreased, the apoptosis rates were significantly increased, and the Ki67 fluorescence intensity was decreased in BC cells. Moreover, caspase 3 was significantly decreased with overexpression of HSPA8 in BC cells. After that, a machine learning prognostic model was created based on the expression of HSPA8 by applying LASSO Cox regression in TCGA and GEO patients. The model indicated that the low‐risk (LR) group with BC had better tumour stages, lymphovascular invasion, and OS than the high‐risk (HR) group. Additionally, the risk score was demonstrated to be an independent risk factor for the prognosis of BC by univariate and multivariate Cox analyses. Moreover, the HR group showed a greater rate of TP53 mutations and was mostly enriched in the ECM‐receptor interaction pathway than the LR group. Importantly, lower CD8^+^ T‐cell and NK cell infiltration, higher immune exclusion scores, higher expression of PD‐L1 and CTLA4 and poorer immune checkpoint therapy effects were found in the HR group. These findings demonstrated how crucial HSPA8 plays a role in determining the prognosis of bladder cancer.

## 1 INTRODUCTION

Bladder cancer (BC) is the 10th most commonly diagnosed cancer globally and is diagnosed in more than 430,000 men and women worldwide each year, with a male to female ratio of approximately 4:1.^1^ Muscle‐invasive bladder cancer (MIBC) is the term for tumours that infiltrate the detrusor and have a higher propensity to migrate to lymph nodes and other organs. Nonmuscle‐invasive bladder cancer (NMIBC) includes distinct entities, including carcinoma in situ (CIS), papillary non‐invasive tumours, and papillary tumours invading the lamina propria.^2^, ^3^ Among them, MIBC accounts for approximately 70% of new patients and is the leading cause of death in patients.^4^ The treatment of bladder cancer includes surgery, chemoradiotherapy, immunotherapy and so on. Molecular analysis of bladder cancer helps to improve our understanding of tumour biology and identify several therapeutic targets, such as programmed death 1 (PD‐1) and programmed cell death ligand 1 (PD‐L1).^5^ Over the past 10 years, therapy options have significantly risen as a result of improved knowledge of the aetiology of bladder cancer and growing interest in clinical drug development for bladder cancer. Ongoing activities focus on improving the use of therapies through the development of predictive biomarkers and rational combination regimens.^4^

Heat shock protein family A member 8 (HSPA8), also known as HSC70, HSC71, HSP71 or HSP73, is the major housekeeping protein of the HSP70 family. It is involved in many functions and is therefore an essential protein.^6^, ^7^ In an ADP/ATP‐dependent manner, HSPA8 binds to tiny hydrophobic regions of developing or partially unfolded proteins. HSPA8 initially binds client proteins in an ATP‐binding state with low affinity and a high rate of exchange. Following this binding, it hydrolyzes ATP to ADP with the aid of auxiliary proteins from the HSP40 family, assuming a high affinity, slow exchange rate ADP‐bound state. By the action of the so‐called nucleotide exchange factor (NEF), it reverts to the ATP state, thereby releasing its substrate peptide.^8^ Yoko Matsuda et al.^9^ showed that altering the posttranslational modification of the heat shock protein HSPA8 can inhibit the stem cell phenotype of glioblastoma. Yufei Wang et al.^10^ suggested that HSPA8 inhibited ferroptosis in HCC cells by upregulating SLC7A11/GPX4 expression and reducing erastin‐mediated accumulation of reactive oxygen species and Fe2+ in cells in vitro and in vivo. Jun Li et al.^11^ showed that high HSPA8 expression predicts poor outcomes in acute myeloid leukaemia. These evidences suggest an important function of HSPA8 in the development of diseases, including cancer. However, the biological function of HSPA8 in bladder cancer remains unclear.

Therefore, this study aims to explore the biological role of HSPA8 in the prognosis of bladder cancer and establish a prognostic risk model based on HSPA8 to predict the overall survival, progression and immune microenvironment of bladder cancer patients. This study is expected to provide new molecular targets for the precise treatment of bladder cancer.

## 2 MATERIALS AND METHODS

### 2.1 Cell line culture

Human bladder cancer cell lines (T24, UMUC3, RT112, 5637) were cultured in Roswell Park Memorial Institute medium‐1640 (RPMI‐1640; Gibco) supplemented with 10% foetal bovine serum (FBS) and 1% penicillin–streptomycin solution (100×; Beyotime). The SVHUC cell line was cultured in F12K medium (Boster) supplemented with 10% FBS and 1% penicillin–streptomycin solution. All cells were cultured at 37°C and 5% CO_2_ in a cell incubator.

### 2.2 Cell transfection

The HSPA8 overexpression plasmid and HSPA8 shRNA plasmid were purchased from GeneCopoeia™. We plated cells into 6‐well plates and waited for cells in the logarithmic growth phase to transfect. Then, we changed the culture medium to 1.5 mL of Opti‐MEM (Gibco) 2 h before transfection. Next, one EP tube contained 245 μL Opti‐MEM and 5 μL Lipo2000 (Invitrogen), and the other EP tube contained 250 μL Opti‐MEM solution with 5 μg plasmid and allowed to stand for 5 min. Furthermore, the solution in two tubes was well mixed and allowed to stand for 10 min to transfect cells by using 500 μL transfection solution in each well. We changed the transfection medium to 2 mL of antibiotic‐free medium after 8 h. Finally, proteins were extracted 48 h after transfection from these transfected cells to examine the transfection effect.

### 2.3 Wound healing assay

A horizontal line was drawn on the back of the 6‐well plate before seeding the cells. The cells in different treatment groups (HSPA8‐overexpressing group and control group) were digested and centrifuged at 200 *g* for 3 min after termination of digestion. The supernatant was removed and rinsed again with PBS, and the cells were counted. After cell counting, the cells were connected to 6‐well plates, and the number was appropriate to cover the bottom of the plate after adherence. After the cells were spread all over the bottom of the plate, a cell scratch was made with a 200‐μL pipette tip perpendicular to the well plate, and the width of each scratch was as consistent as possible. The cell culture medium was aspirated off, and the wells were washed three times with PBS to wash away cell debris generated by the scratch. Serum‐free medium was added, and photographs were taken for records. The plates were incubated in an incubator and photographed after 24 h.

### 2.4 Western blotting

The primary antibodies used in this study included anti‐HSC70 (ab51052; Abcam), anti‐caspase 3 (ab32351; Abcam) and anti‐beta actin (ab8226; Abcam). First, the proteins of bladder cancer cells were extracted, and the protein concentration was determined. Then, the proteins of each group were electrophoresed under the conditions of a constant pressure of 100 V and stopped when the dye front moved 2–3 mm from the bottom of the gel (approximately 120 min). Subsequently, we transferred the proteins on the gel to the FVDF membrane under the following conditions: constant current 200 mA for 2 h. Next, we added 5% skim milk to the PVDF membrane containing protein and blocked it at room temperature for 1 h. The sealed PVDF membrane was incubated with primary antibody and then placed in a 4°C refrigerator overnight. The next day, the primary antibody was recovered, and the secondary antibody was added and incubated at room temperature for 1 h. Scanning densitometry was performed with ImageJ software (V1.53), and GraphPad Prism software (V9.4.1) was used to generate statistical graphs.

### 2.5 Flow cytometry

Flow cytometry was processed by following the protocol of the Annexin V‐FITC/PI apoptosis kit (AP101; Multi Science) and using a flow cytometry system. The experiment was repeated at least three times.

### 2.6 Immunofluorescence

Cells from each group (10^6^–10^7^ in total) were collected in 2 mL ep tubes, added to 1.8 mL PBS, and then gently reversed up and down several times. After centrifugation at 800× *g* for 5 min × 3 times, the supernatant was discarded. One millilitre of 4% paraformaldehyde was added and placed at room temperature for 10 min. The tube base was flicked several times at an interval of 2 min to make the cells fully contact the solution. The samples were centrifuged at 800× *g* for 5 min × 3 times, and the supernatant was discarded. The cells were blocked and permeabilized by adding 1 mL of 1% BSA + 0.4% Triton‐X‐100. The bottom of the tube was flicked several times at an interval of 5 min to make the cells fully contact the solution. The samples were centrifuged at 800× *g* for 5 min × 3 times, and the supernatant was discarded. One millilitre of diluted primary antibody was added, left at room temperature for 2 h, and centrifuged at 800× *g* for 5 min, and the supernatant was discarded. One millilitre of secondary antibody with fluorescent labelling was added in the dark, placed at room temperature for 1 h, and centrifuged at 800× *g* for 5 min, and the supernatant was discarded. One to two drops of DAPI solution and 10 μL of PBS were added and placed in the dark at room temperature for 5 min. Cells were dropped onto slides (1–2 drops) using a 10 μL pipetter, allowed to dry slightly, and photographed under a microscope behind the coverslips.

### 2.7 Apoptosis fluorescence

The apoptosis fluorescence was processed by following the protocol of the Annexin V‐FITC/PI apoptosis kit (AP101; Multi Science) and DAPI solution, which was photographed by an immunofluorescence imaging system.

### 2.8 TCGA and GEO data acquisition and preprocessing

The transcriptomics, SNP data and related clinical information of 411 BCs were obtained from the TCGA database (https://portal.gdc.cancer.gov/repository). For transcriptome and clinical data, the raw data for each case was downloaded separately. We use Perl software to extract information from each case and merge it into a complete matrix file. The gene names of the merged transcriptome matrix were annotated with annotation files, removing data with excessive missing values (>50%) and standardising these data using log2 (*x* + 1) for subsequent analysis. The merged clinical information requires removal of unnecessary clinical parameters and missing information and matching of patient ID with transcriptome data for subsequent analysis. The downloaded SNP data can be directly used after matching with the patient ID of the transcriptome data. The transcriptomics data and related clinical information of 165 BC patients were obtained from the GSE13507 dataset (https://www.ncbi.nlm.nih.gov/geo/query/acc.cgi?acc=GSE13507), and 308 BC patients were obtained from GSE32894 dataset (https://www.ncbi.nlm.nih.gov/geo/query/acc.cgi?acc=GSE32894). The downloaded transcriptome data and clinical information exist in the same matrix file, and we separated the transcriptome data and clinical information from this matrix. Transcriptome data were annotated using platform files (GPL6102 for the GSE13507 dataset and GPL6947 for the GSE32894 dataset), deleting data with excessive missing data (>50%) and standardising these data using log2 (*x* + 1) for subsequent analysis. The clinical information requires removal of unnecessary clinical parameters and missing information and matching of patient ID with transcriptome data for subsequent analysis.

### 2.9 Identification of differentially expressed genes

The differentially expressed genes (DEGs) of the HSPA8‐high group and HSPA8‐low group were finally identified by applying the edge R package with FDR < 0.05 and |log2FC| ≥ 0.585 in both TCGA and GEO cohorts.

### 2.10 Establishment of the machine learning prognostic model

We established a machine learning prognostic model by applying LASSO Cox regression analysis in both the TCGA and GSE13507 datasets, and 20 overall survival‐related genes were finally included in our model. In our work, the risk score was calculated by $\sum_{i}^{8}A_{i}\times B_{i}$ (*A*: coefficients, *B*: gene expression level), and the patients were divided into a high‐risk group and a low‐risk group based on the risk scores for further study.

### 2.11 Functional enrichment analysis

In this work, we first performed gene differential analysis of the HR group and LR group in the TCGA and GSE13507 datasets and identified 159 and 274 significantly different genes in the TCGA dataset and GSE13507 dataset, respectively. These genes were used for the Kyoto Encyclopaedia of Genes and Genomes (KEGG) enrichment analysis. For gene set enrichment analysis (GSEA), TCGA and GSE13507 transcriptome data and risk files were input into R software. The programme performs enrichment analysis by sorting the expression profile data, calculating enrichment scores, estimating significance levels and performing multiple hypothesis testing.

### 2.12 Statistical analysis

One‐way anova and *t* tests were used to compare two groups, and the comparisons for two or more component percent rates were made using the chi‐square test. We processed all statistical analyses by utilising SPSS 19.0 software (SPSS Inc.) or R software. In our study, a *p* value <0.05 was statistically significant.

## 3 RESULTS

### 3.1 HSPA8 was closely associated with poor prognosis of BC patients

**FIGURE 1:**
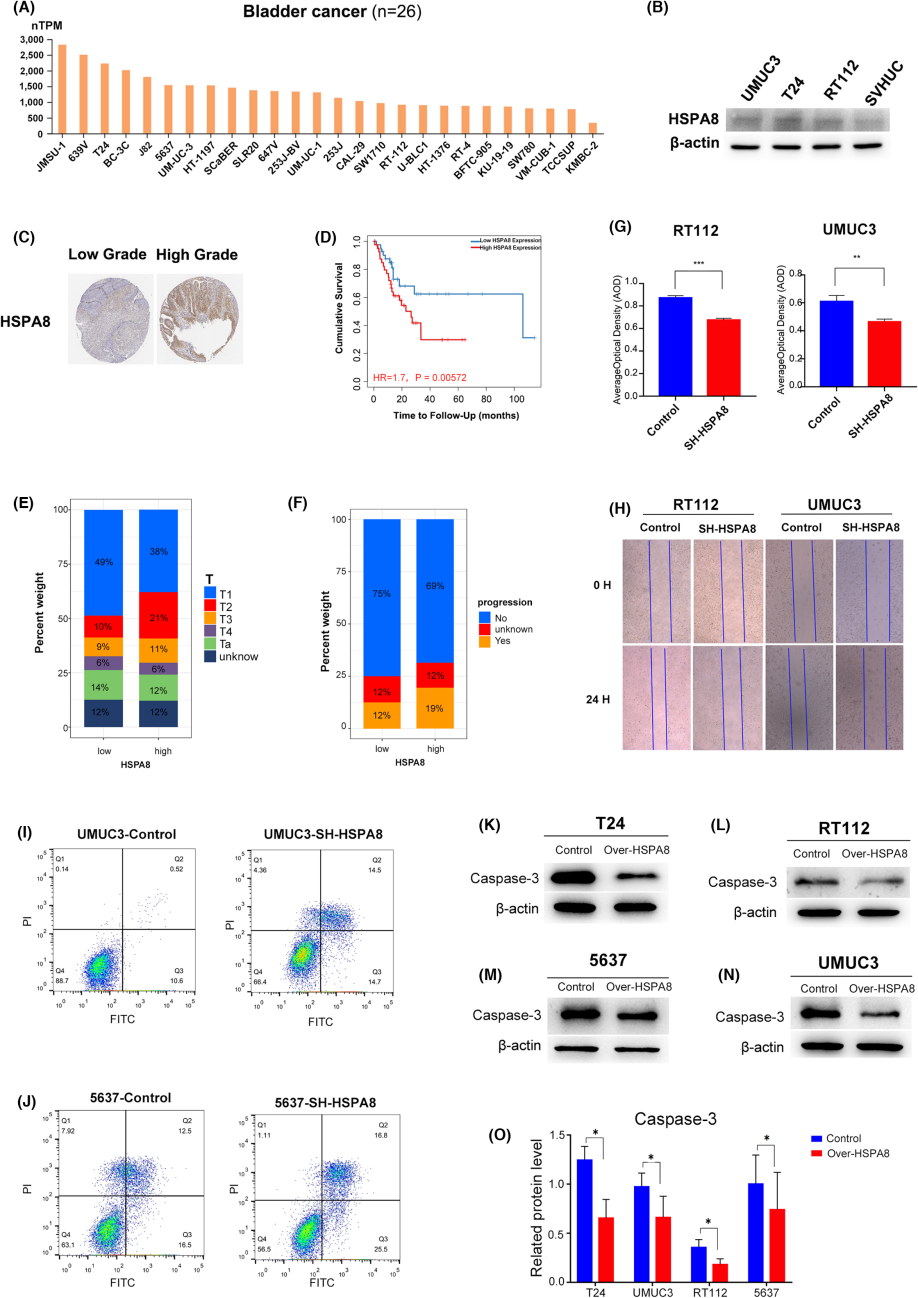
HSPA8 was closely associated with poor prognosis of BC patients. (A) HPA database analysis of the gene expression of HSPA8 in bladder cancer cell lines. (B) HSPA8 protein expression in both normal bladder cells and bladder cancer cell lines. (C) The HPA database reports that HSPA8 protein is expressed in both low‐grade and high‐grade bladder cancer tissues. (D) Comparison of overall survival rates between TCGA patients in the HSPA8‐high and HSPA8‐low groups. (E) The percentage weight of T stages among TCGA patients in the HSPA8‐high and HSPA8‐low groups. (F) Progression weight in TCGA patients in the HSPA8‐high and HSPA8‐low groups. (G) CCK8 test of UMUC3 and RT112 cells in the SH‐HSPA8 and control groups. (H) Wound healing assays were used to detect the migration of UMUC3 and RT112 cells in the control group and SH‐HSPA8 group. (I, J) Flow cytometry of UMUC3 and 5637 cells in the control group and SH‐HSPA8 group. (K–O) Caspase 3 protein expression levels of the control group and over‐HSPA8 group in T24, RT112, 5637 and UMUC3 cells.

First, we examined the expression of HSPA8 in bladder cancer cell lines in the HPA database. Notably, all bladder cancer cell lines expressed HSPA8 at high levels, and the expression levels of muscle‐invasive BC cell lines (e.g., T24, UMUC3 and HT‐1197) were significantly higher than those of nonmuscle‐invasive BC cell lines (e.g., RT112 and RT‐4; Figure 1A). Additionally, we discovered that BC cells (T24, UMUC3 and RT112) expressed HSPA8 at a level that was considerably higher than that of normal bladder cells (SVHUC; Figure 1B). Next, we observed in the HPA database that high‐grade BC tissues expressed more HSPA8 than low‐grade BC tissues (Figure 1C). In addition, based on the TCGA database, we noticed that the HSPA8‐high group had worse overall survival than the HSPA8‐low group and that the percent weight of pathologic T2–T4 in the HSPA8‐high group was higher than that in the HSPA8‐low group (38% vs. 25%; Figure 1D,E). The percent weight of progression in the HSPA8‐high group was higher than that in the HSPA8‐low group (19% vs. 12%; Figure 1F). These findings imply that the poor prognosis of BC patients may be associated with HSPA8. Next, we reduced the amount of HSPA8 expression in the UMUC3 and RT112 cell lines, and the results revealed that bladder cancer cells' capacity for proliferation and migration was dramatically reduced. (Figure 1G,H). In addition, we found that the proportion of early and late apoptotic cells was significantly increased with HSPA8 knockdown in UMUC3 and 5637 cells (Figure 1I,J). Similarly, apoptosis fluorescence also showed that the intensity of early apoptosis fluorescence (FITC) and late apoptosis fluorescence (PI) was significantly enhanced with HSPA8 knockdown in T24 and RT112 cells (Figure S1A). Furthermore, we discovered that overexpression of HSPA8 in the bladder cancer cell lines T24, UMUC3, 5637, and RT112 dramatically reduced the protein expression of caspase‐3. (Figure 1K–O). Immunofluorescence showed that the fluorescence intensity of Ki67 was significantly reduced when we knocked down HSPA8 gene expression in T24 and RT112 cells (Figure S1B). These evidences suggest that HSPA8 is closely related to the poor prognosis of BC.

### 3.2 A machine learning prognostic model based on HSPA8 was established for BC patients

**FIGURE 2:**
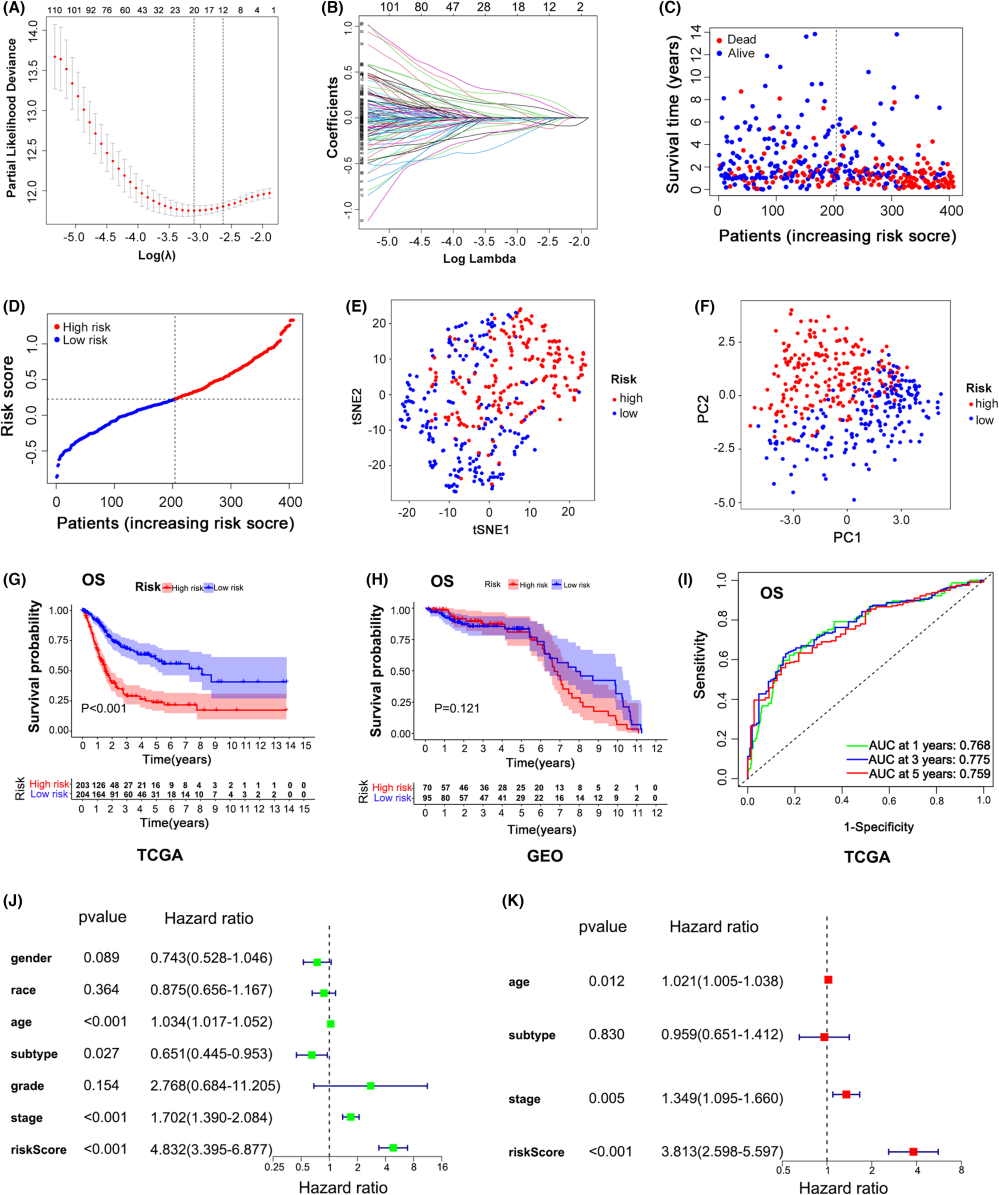
A prognostic risk model based on HSPA8 was established for BC patients. (A,B) LASSO Cox regression analysis for BC patients. (C) The survival status and risk scores of patients in TCGA. (D) The TCGA patient risk curve. (E,F) TCGA patient analysis using tSNE and PC. (G,H) Analysis of overall survival for patients in the TCGA or GEO who were in the HR or LR groups. (I) ROC analysis of the accuracy of the risk score as a predictor of overall survival. (J,K) Clinical parameters and risk scores of TCGA patients were analysed using univariate and multivariate methods.

Based on the TCGA cohort, we discovered 706 genes that were differentially expressed between the HSPA8‐high group and the HSPA8‐low group. And 145 of these genes were shown to be highly associated with overall survival. Next, we established a machine learning prognostic model based on these genes by applying LASSO Cox regression (Figure 2A,B) in the TCGA cohort. By cross‐checking the GSE13507 dataset, 20 overall survival‐related genes were finally included in our model. In addition, we, respectively calculated the risk score of these 20 overall survival‐related genes by running the formula: Risk score = (0.0085 × expression of MT1A) + (−0.0182 × expression of TBX1) + (−0.0070 × expression of UBD) + (0.0644 × expression of AIFM3) + (−0.1426 × expression of PTPRR) + (−0.1602 × expression of AMY2B) + (−0.0030 × expression of SLC23A3) + (0.2692 × expression of MAP2) + (−0.2480 × expression of GNLY) + (0.1463 × expression of SERPINB2) + (0.1231 × expression of ADAMTS15) + (0.1020 × expression of POLR3G) + (0.1241 × expression of SLC1A6) + (0.2731 × expression of ALDH1L2) + (−0.1333 × expression of CD3G) + (0.0864 × expression of PCDHB8) + (−0.0291 × expression of POU5F1) + (−0.0529 × expression of ZNF683) + (−0.0081 × expression of CTSE) + (−0.0688 × expression of PPP2R2B). We observed that as the risk score increased, the number of fatalities increased gradually and that the overall survival time was often shorter in the group with higher risk scores (Figure 2C). Accordingly, we separated the patients into two groups: high‐risk (HR) and low‐risk (LR) groups based on the mean value of the risk score (Figure 2D). According to PC analysis and tSNE analysis, the HR and LR groups could be well distinguished into two groups (Figure 2E,F). The findings revealed that in both the TCGA and GEO cohorts, the HR group had a lower overall survival probability than the LR group. (Figure 2G,H). ROC analysis was used to demonstrate our model's capacity to predict overall survival, and the areas under the ROC curve (AUC) for 1‐, 3‐ and 5‐year survival were 0.768, 0.775 and 0.759, respectively. (Figure 2I). Besides, both univariate and multivariate analyses revealed that the risk score was an independent risk indicator for BC patients. (Figure 2J,K). These evidences indicate that the risk model is reasonable and can accurately predict the prognosis of BC patients.

### 3.3 Gene mutation and functional enrichment analysis for the two risk groups

**FIGURE 3:**
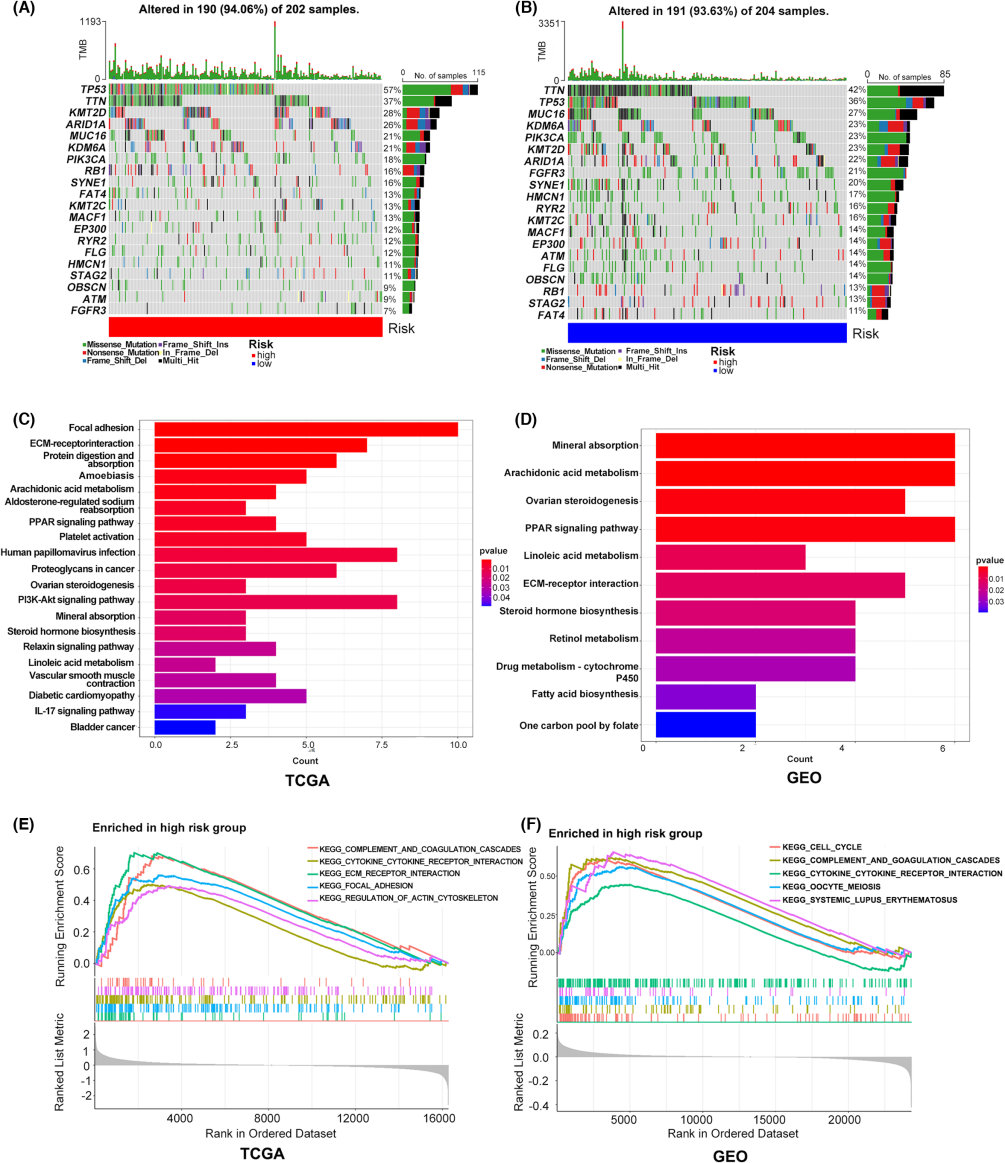
Gene mutation and functional enrichment analysis for the two risk groups. (A,B) Gene mutation status for the HR group and LR group. (C,D) KEGG enrichment analysis for genes with differential expression between the HR group and LR group in TCGA or GEO patients. (E,F) GSEA enrichment analysis for patients in the TCGA or GEO database who are in the HR group.

We utilised Maftools to find entire gene mutations in 406 BC patients with complete overall survival data from the TCGA cohort to compare the genetic mutation status between the two risk categories. (Figure 3A,B). Similar mutation frequencies were found in the HR and LR groups (94.06% vs. 93.63%), but the HR group had more mutations in genes such as TP53, KMT2D, and ARID1A than the LR group (57% vs. 36%, 28% vs. 23% and 26% vs. 22%), which could be attributed to genetic heterogeneity. These altered genes might help explain some of the poor clinical prognosis for BC in the HR group.

We discovered that the differentially expressed genes in the TCGA cohort between the HR group and LR group were primarily enriched in the ECM‐receptor interaction, PPAR signalling pathway, PI3K‐Akt signalling pathway, proteoglycans in cancer and bladder cancer pathway. Additionally, GSEA enrichment analysis revealed that, in comparison to the LR group, the HR group was primarily enriched in the ECM‐receptor interaction, focal adhesion and cytokine receptor interaction pathways. (Figure 3C,E). In the GEO cohort, we also discovered that the ECM‐receptor interaction and PPAR signalling pathways were primarily enriched in the differentially expressed genes between the HR group and the LR group, and GSEA revealed that the HR group was more significantly enriched in the cytokine receptor interaction pathways than the LR group. (Figure 3D,F). These findings highlighted a possible reason for the poorer prognosis of the HR group, and the route involving the ECM and receptor interactions may be essential to this process.

### 3.4 The HR risk group predicts poor clinical characteristics and survival of BC patients

**FIGURE 4:**
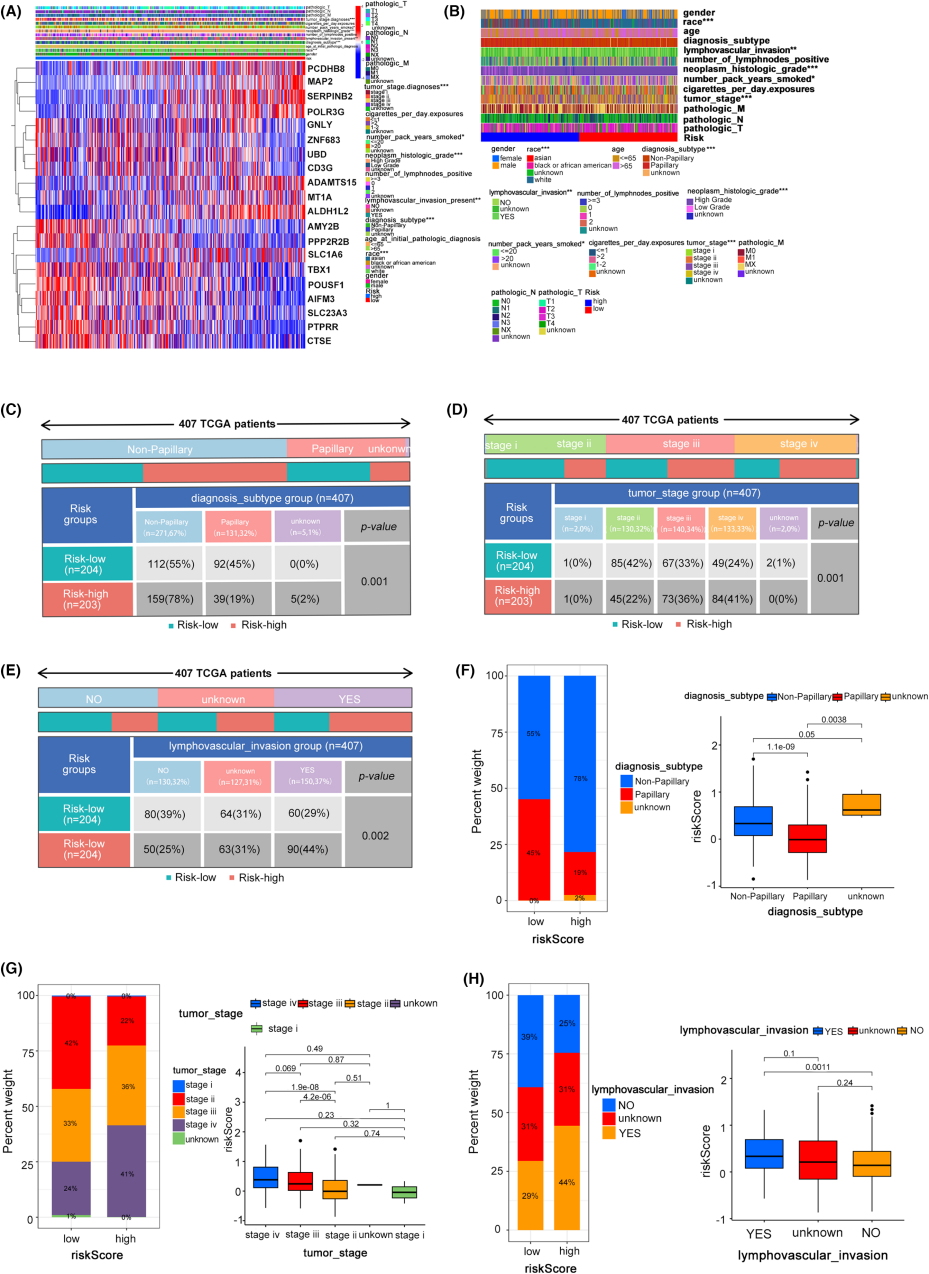
The HR risk group predicts poor clinical characteristics and survival of BC patients. (A) Gene expression levels of the constructed model among the HR group and LR group in TCGA patients. (B) *T* test for clinical parameters (tumour stage, diagnosis subtype, lymph node invasion, etc.) among the HR group and LR group in TCGA patients. (C–E) Chi‐square test for tumour stage, diagnosis subtype and lymph node invasion among the HR group and LR group in TCGA patients. (F–H) Percent weight and box analysis for tumour stage, diagnosis subtype, and lymph node invasion among the HR group and LR group in TCGA patients.

We noticed that the 20 genes utilised to build the model in the TCGA cohort were significantly different between the HR group and the LR group (Figure 4A). Additionally, the results of the *T* test and chi‐square test revealed that there were significant differences between the HR group and LR group in terms of the bladder cancer patients' diagnostic subtype, lymph node invasion, and tumour stage (Figure 4B–E). Patients in the HR group had more nonpapillary BC than those in the LR group in terms of the diagnostic subtype (78% vs. 55%). In other words, patients with nonpapillary BC had greater risk scores than those with papillary BC (Figure 4C,F). In terms of lymph node invasion, the HR group had more patients who had lymph node invasion (44% vs. 29%) than the LR group. In other words, patients with lymph node invasion had higher risk scores than those without lymph node invasion (Figure 4E,H). Regarding tumour stage, the HR group had more patients with bladder cancer at stages 3–4 (77% vs. 57%) than the LR group. In other words, patients with high‐grade bladder cancer had higher risk scores than low‐grade patients (Figure 4D,G). In both the GSE13507 and GSE32894 dataset, we found that these 20 genes used to construct the model were also significantly different in the HR group and LR group, and the gene expression patterns are consistent with TCGA cohort. (Figures S2A and S3A). In the GSE13507 dataset, the *T* test and chi‐square test findings demonstrated a significant difference between the HR group and LR group in terms of the invasiveness, *T* stage, and tumour grade of BC patients. (Figure S2B–E). For invasiveness, the HR group had a higher proportion of muscle‐invasive bladder cancer than the LR group (59% vs. 22%). In other words, patients with muscle‐invasive BC had higher risk scores than those with nonmuscle‐invasive BC (Figure S2D,G). For T stage, the HR group had a higher proportion of T3–T4 patients than the LR group (26% vs. 12%). In other words, T3–T4 patients had higher risk scores than Ta–T2 patients (Figure S2E,H). Regarding tumour grade, the HR group had a greater percentage of patients with high‐grade BC than the LR group (59% vs. 20%), which means that high‐grade BC patients had higher risk scores than low‐grade patients (Figure S2C,F). To obtain more reliable results, we further verified it on the GSE32894 dataset, and the results of the *T* test and chi‐square test also showed a significant difference between the HR group and LR group in terms of the tumour stage and tumour grade of BC patients (Figure S3B–D). For tumour stage, the HR group had a higher proportion of T2–T4 patients than the LR group (41% vs. 21%), which means that T2–T4 patients had higher risk scores than Ta–T2 patients (Figure S3C,E). Similarly, the HR group had a greater percentage of high‐grade (G3–G4) patients than the LR group (61% vs. 42%), which means that high‐grade patients had higher risk scores than low‐grade (G1–G2) patients (Figure S3D,F).

**FIGURE 5:**
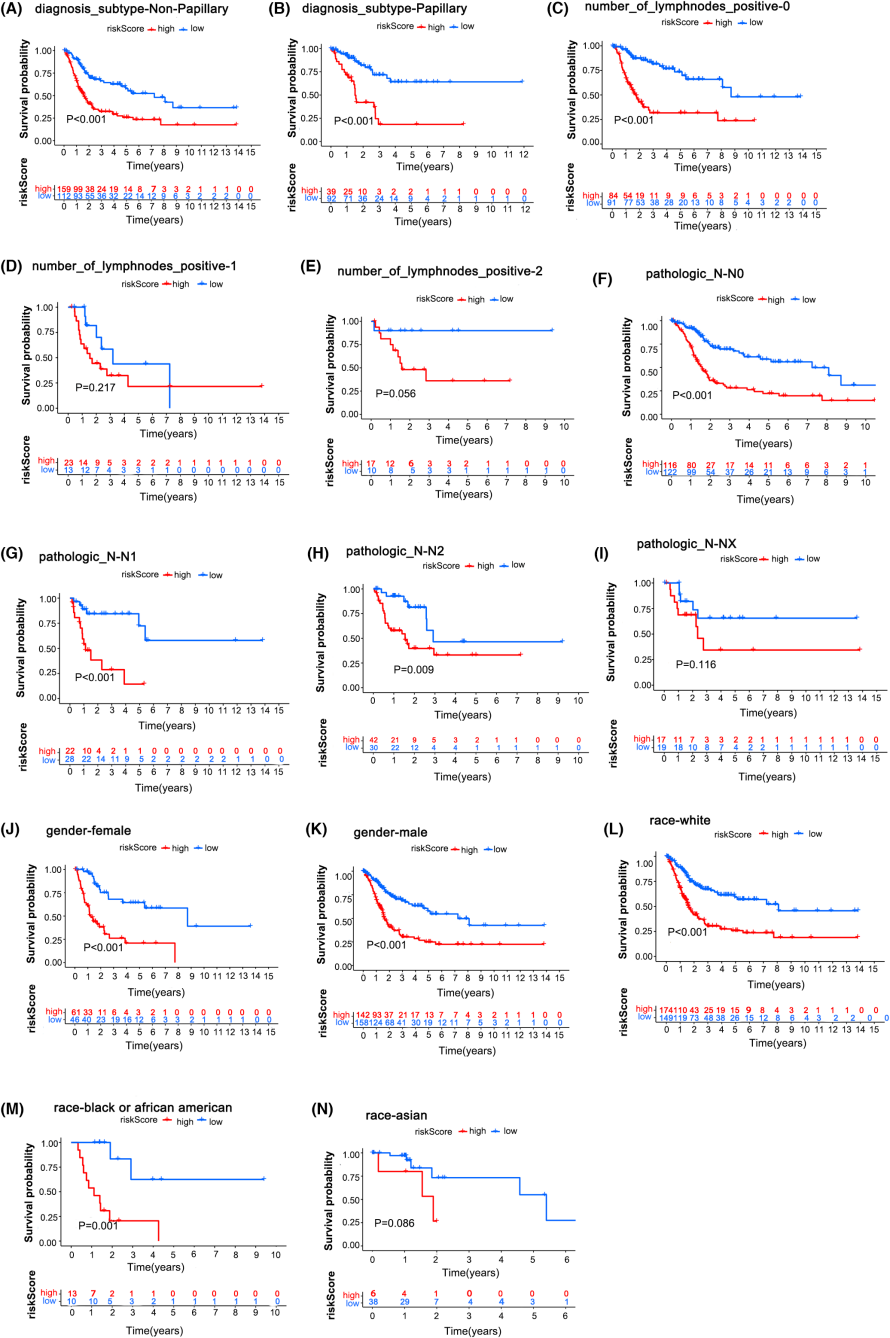
The HR risk group predicts poor clinical characteristics and survival of BC patients. (A–N) Overall survival analysis for the HR group and LR group in different clinical subgroups of TCGA patients.

Moreover, we discovered that the overall survival of the HR group was poorer than that of the LR group in many clinical subgroups (such as race, diagnostic subtype, tumour stage, etc.) in both the TCGA and GEO cohorts. The details are shown in Figure 5 and Figure S4. These findings revealed that, compared to the LR risk group, the HR risk group better predicted the clinical features and survival of BC patients.

### 3.5 The HR group predicted a higher risk of immune escape for BC patients

**FIGURE 6:**
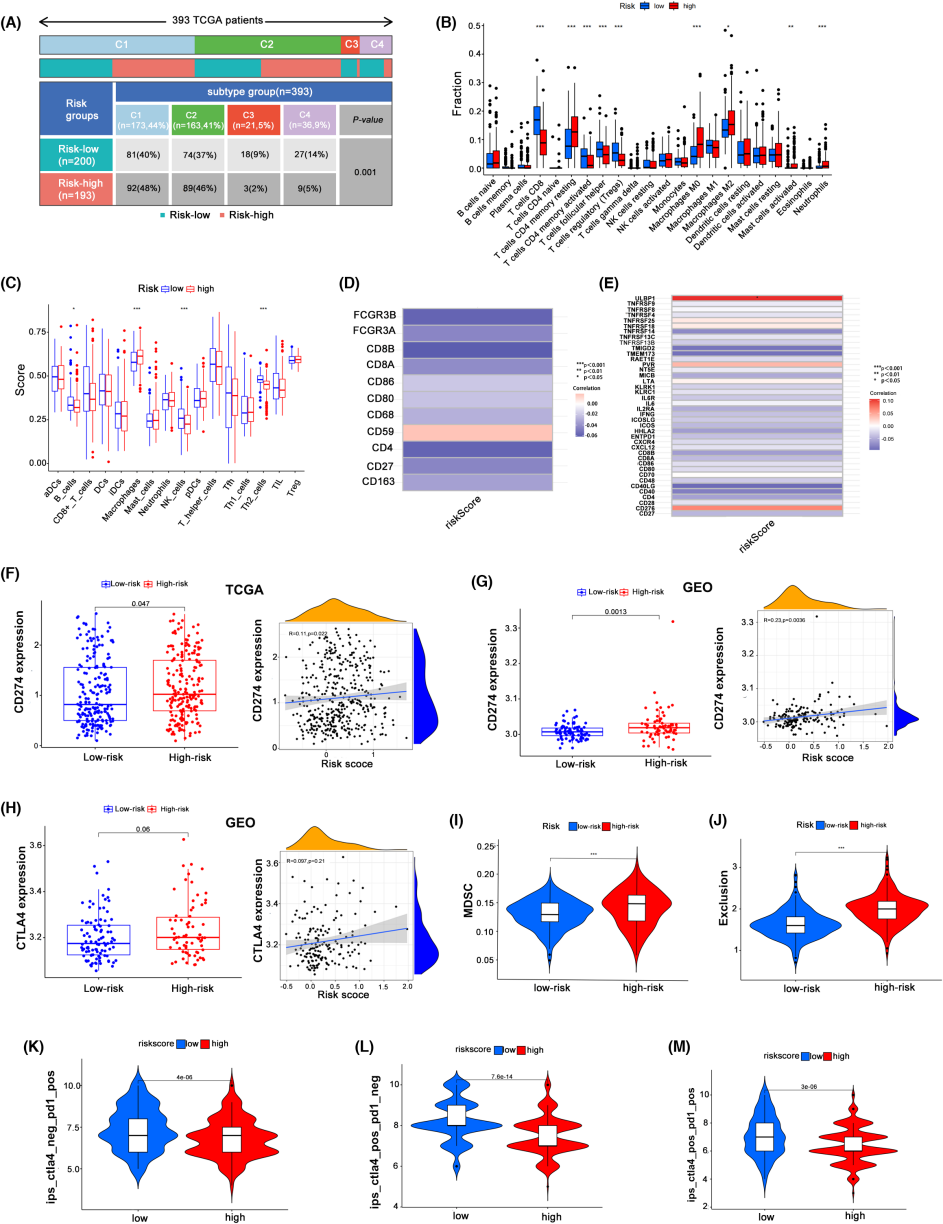
The HR group predicted a higher risk of immune escape for BC patients. (A) Chi‐square test for immunological subtypes (C1–C4) in the TCGA patients' HR and LR groups. (B,C) Analysis of immune infiltration for the HR group and LR group of TCGA patients using ssGSEA and Cybersort. (D,E) Correlation study of immunological marker genes and immune activation genes with the risk score. (F) Correlation analysis between CD274 and the risk score in TCGA patients and differences in CD274 expression between the HR group and LR group. (G) Correlation analysis between CD274 and risk score in GEO patients and differences in CD274 expression between the HR group and LR group. (H) In TCGA patients, the correlations between CTLA‐4 and the risk score and the difference in CTLA‐4 expression between the HR group and LR group. (I) MDSC expression variations in the TCGA patients' HR and LR groups. (J) Variations in immune exclusion ratings between the HR and LR groups of TCGA patients. (K) Negative CTLA‐4 and positive PD1 immunotherapy effect between the HR and LR groups. (L) The immunotherapy effects between the HR and LR groups were positive for CTLA‐4 and negative for PD1. (M) Positive CTLA‐4 and positive PD1 immunotherapy effect between the HR and LR groups.

First, we discovered a definite difference in immunological subtypes between the HR and LR groups. In particular, wound healing (Immune C1) and IFN‐gamma dominant (Immune C2) had higher proportions in the LR group (23% vs. 7%), whereas inflammatory (Immune C3) and lymphocyte depleted (Immune C4) had higher proportions in the HR group (94% vs. 77%; *p* = 0.001), which showed the role of risk groups in differentiating immunity. (Figure 6A). Then, ssGSEA and CIBERSORT immune infiltration analyses revealed that the degree of infiltration of functional immune cells, such as CD8+ T cells, B cells and NK cells, was lower in the HR group than in the LR group. (Figure 6B,C). Furthermore, in bladder cancer patients, risk scores were inversely linked with the expression levels of immune marker genes (CD8A, CD80, etc.) and immune activation‐related genes (IL6, IFNG, etc.; Figure 6D,E). According to the TCGA or GEO databases, the HR group also showed higher immunological checkpoint (CD274, CTLA‐4) expression than the LR group (Figure 6F,G). Furthermore, patients in the HR group showed greater immune rejection scores and MDSC expression levels than those in the LR group (Figure 6I,J). These data show that patients in the HR group are more likely than those in the LR group to experience immunological escape. Finally, we projected how patients in the HR and LR groups would respond to immunotherapy. The results showed that when patients received PD‐1‐ or CTLA‐4‐positive immunotherapy, the LR group achieved a better therapeutic effect than the HR group (Figure 6K–M).

## 4 DISCUSSION

Heat shock proteins (HSPs), a broad family of proteins involved in protein folding and maturation, are triggered by heat shock or other stresses. Their components mainly include HSP27, HSP40, HSP60, HSP70, HSP90, etc.^12^ It has been reported that these proteins are highly expressed in a series of cancers, such as breast, prostate, colorectal, lung, ovarian, gastric, oral and oesophageal cancers, and their expression contributes to cancer proliferation, invasion and metastasis.^13^ Detection of serum levels of heat shock proteins and their specific antibodies in affected individuals plays an important role in cancer diagnosis.^13^ Heat shock protein family A member 8 (HSPA8) is a member of the HSP70 family. HSPA8 protein is only 25% different from Hsp70 in amino acid sequence, whose major localization is in the cytoplasm, and has been shown to support a variety of oncogenic processes by regulating client proteins.^14^ For instance, HSPA8 supports the assembly and activity of the cyclin D1 holoenzyme by binding and folding freshly generated cyclin D1, and cyclin D1 is amplified and overexpressed in a variety of malignancies, where it speeds up the proliferation of cancer cells.^15^, ^16^ These data reveal that HSPA8 is a key player in the development of cancer. Nevertheless, the biological significance of HSPA8 in bladder cancer needs to be clarified. Our research proved that HSPA8 facilitated metastasis, blocked apoptosis and was linked to a poor prognosis for bladder cancer patients. HSPA8 is anticipated to be a novel prognostic molecular target for bladder cancer.

We created a risk model based on HSPA8 to further investigate the predictive usefulness of HSPA8 for the prognosis of BC patients. Notably, the HR group had a markedly enhanced ECM‐receptor interaction pathway. The extracellular matrix (ECM) is an intricate network of macromolecules that provides a suitable place for cell survival and activity and affects cell shape, metabolism, function, migration, proliferation and differentiation through signal transduction systems.^17^, ^18^, ^19^, ^20^ It has been reported that the ECM‐receptor interaction pathway plays a crucial role in the development and progression of breast and colorectal cancer.^21^, ^22^ As a result, it is quite likely that the ECM‐receptor interaction pathway is the underlying molecular cause of the poor prognosis of the HR group. In addition, genetic variants in the tumour suppressor gene TP53 contribute to human cancers in different ways.^23^ The antiproliferative role of the P53 protein in response to various stresses and physiological processes, such as ageing, makes it a major target for cancer inactivation.^24^ The dominant mode of TP53 inactivation is single‐base substitution and loss of alleles, with inactivation of viral or cellular proteins playing a major role in specific cancers.^25^ Moreover, the inheritance of TP53 mutations leads to a predisposition to early‐onset cancers, including breast cancer, sarcoma, brain tumours and adrenocortical carcinomas.^26^ In our research, we discovered that the HR group had a considerably higher prevalence of TP53 mutations than the LR group. TP53 mutation may therefore be another factor contributing to the poor prognosis of the HR group.

Immune cells, as an integral part of the tumour microenvironment (TME), play a crucial role in tumour progression or prognosis.^27^, ^28^ In our research, we observed that the HR group had considerably lower CD8+ T cell, NK cell and B‐cell infiltration levels than the LR group. These evidences suggest another reason for the poor prognosis of the HR group. In addition, tumour immune escape has been considered another major cause of cancer metastasis or progression in recent years.^29^ Its mechanism mainly involves low infiltration of immune cells and high expression of immunosuppressive genes or immune checkpoint genes (PD‐1, PD‐L1, etc.).^30^, ^31^ In contrast to those in the LR group, patients in the HR group exhibited greater immune exclusion ratings, higher expression of immune checkpoint genes (CD274 and CTLA‐4) and lower immune cell infiltration, according to our study. These data provide evidence from a different angle for the poor prognosis of patients in the HR group and show that patients in the HR group may have a higher probability of immunological escape.

Our study revealed a critical role of HSPA8 or its risk model in bladder cancer growth, metastasis, apoptosis, gene mutations, and the tumour immune microenvironment. These findings suggest the feasibility of HSPA8 as a prognostic molecular marker for bladder cancer. However, there are some limitations to our study. First, the HSPA8‐based risk model was constructed or validated in a total of 884 cases from the TCGA dataset, GSE13507 dataset and GSE32894 dataset. Although the current number of cases was relatively large, these datasets were already the better choices among the available datasets with complete transcriptome data and clinical data that can be matched. However, to further strengthen the general applicability of the risk model, it is obvious that these cases are not sufficient in a multiethnic, large‐scale population worldwide. Therefore, it is necessary to explore new datasets or prospectively enrol patients in our centre to further validate the risk model in the future. In addition, this study aimed to focus on the biological function of HSPA8 in bladder cancer, and the deep mechanisms of how HSPA8 exerts these biological functions were not revealed in detail. However, we found that the HR group was significantly enriched in ECM receiver interaction, focal adhesion, cell cycle and cytokine receiver interaction pathways compared with the LR group through functional enrichment analysis; these pathways play a crucial role in disease progression,^21^, ^22^, ^32^, ^33^, ^34^ which may be the potential mechanisms by which HSPA8 exerts its biological functions. Verification of these mechanisms or further exploration of the detailed mechanism of HSPA8 in bladder cancer will be the focus of our next work.

## 5 CONCLUSION

In summary, we indicated that HSPA8 is a potential marker for poor prognosis in bladder cancer. The HSPA8‐based risk model can accurately predict gene mutations, survival, clinical features and immune escape in bladder cancer.

## AUTHOR CONTRIBUTIONS

**Yang Liu:** Data curation (equal); formal analysis (equal); investigation (equal); methodology (equal); validation (equal); writing – review and editing (equal). **Zhong‐qi Pang:** Formal analysis (equal); investigation (equal); methodology (equal); validation (equal); writing – original draft (equal). **Jian‐she Wang:** Formal analysis (equal); investigation (equal); methodology (equal); validation (equal); writing – original draft (equal). **Jing‐feng Wang:** Investigation (equal); methodology (equal); software (equal); validation (equal). **Jia‐xin He:** Data curation (equal); formal analysis (equal); investigation (equal); methodology (equal). **Bo Ji:** Data curation (equal); investigation (equal); methodology (equal). **Lu Zhang:** Data curation (equal); investigation (equal); methodology (equal). **Ming‐hua Ren:** Funding acquisition (equal); investigation (equal); methodology (equal); project administration (equal); supervision (equal); writing – review and editing (equal).

## FUNDING INFORMATION

The Horizontal Project of Heilongjiang Renxin Medical Assistance Foundation, Heilongjiang Charity Fund General Association and Heilongjiang Province Natural Science Foundation (No. LH2019H030 to Ming‐hua Ren) provided funding for this study.

## CONFLICT OF INTEREST STATEMENT

The authors declare that they have no conflicts of interest.

## Supporting information


Figure S1

Figure S2

Figure S3

Figure S4

## Data Availability

The TCGA data portal (https://tcga‐data.nci.nih.gov/tcga/) and the GEO data portal (http://www.ncbi.nlm.nih.gov/geo/) both contain all of the data used in this investigation.
