# Supplementary figures and images for: Heat shock protein family A member 8 is a prognostic marker for bladder cancer: Evidences based on experiments and machine learning
Source: J Cell Mol Med. 2023 Sep 28;27(24):3995–4008. doi: 10.1111/jcmm.17977 (PMC10746959; doi:10.1111/jcmm.17977)

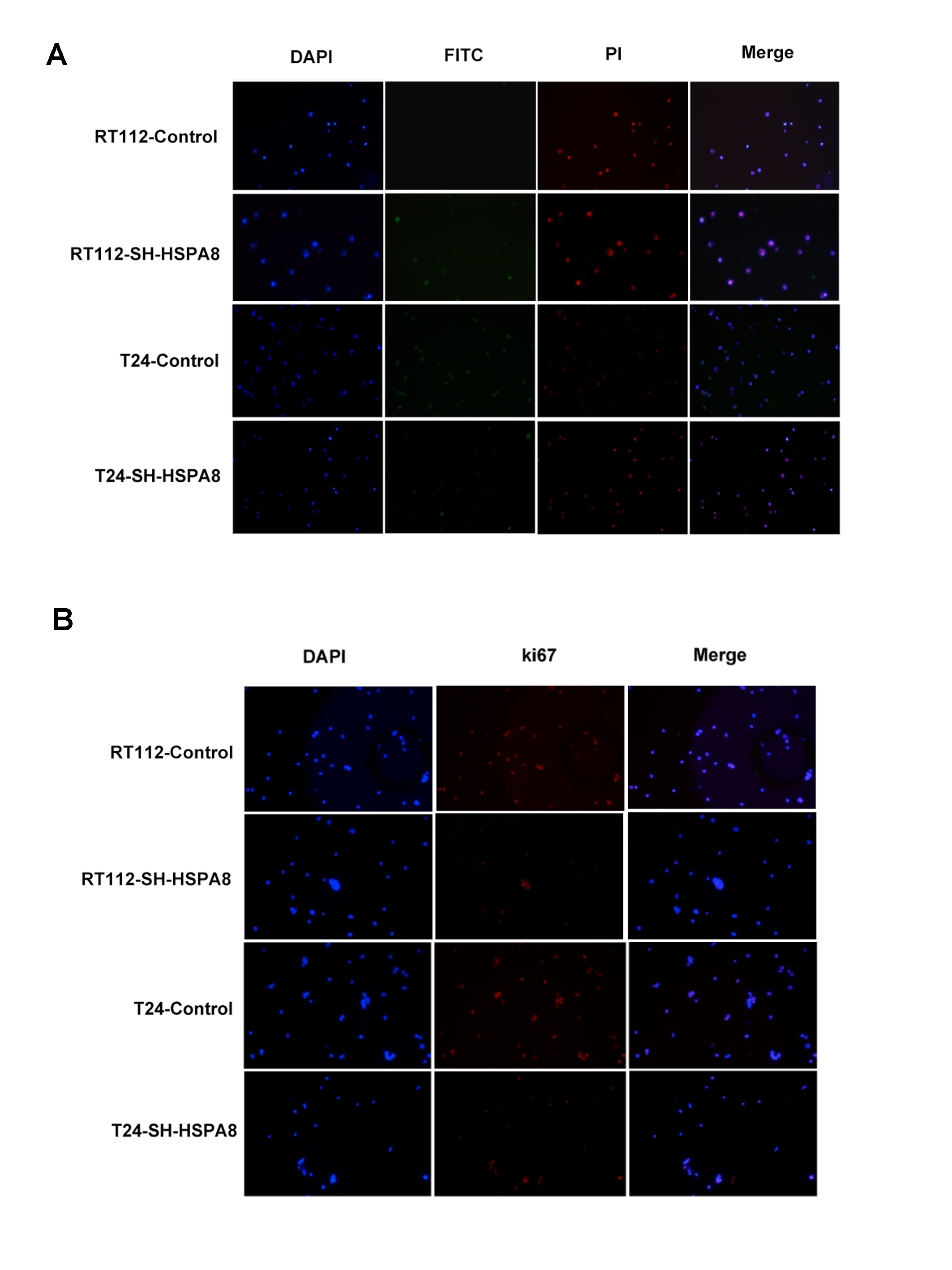

Supplement: Supplementary file 1 — Figure S1 [file JCMM-27-3995-s004.tif]

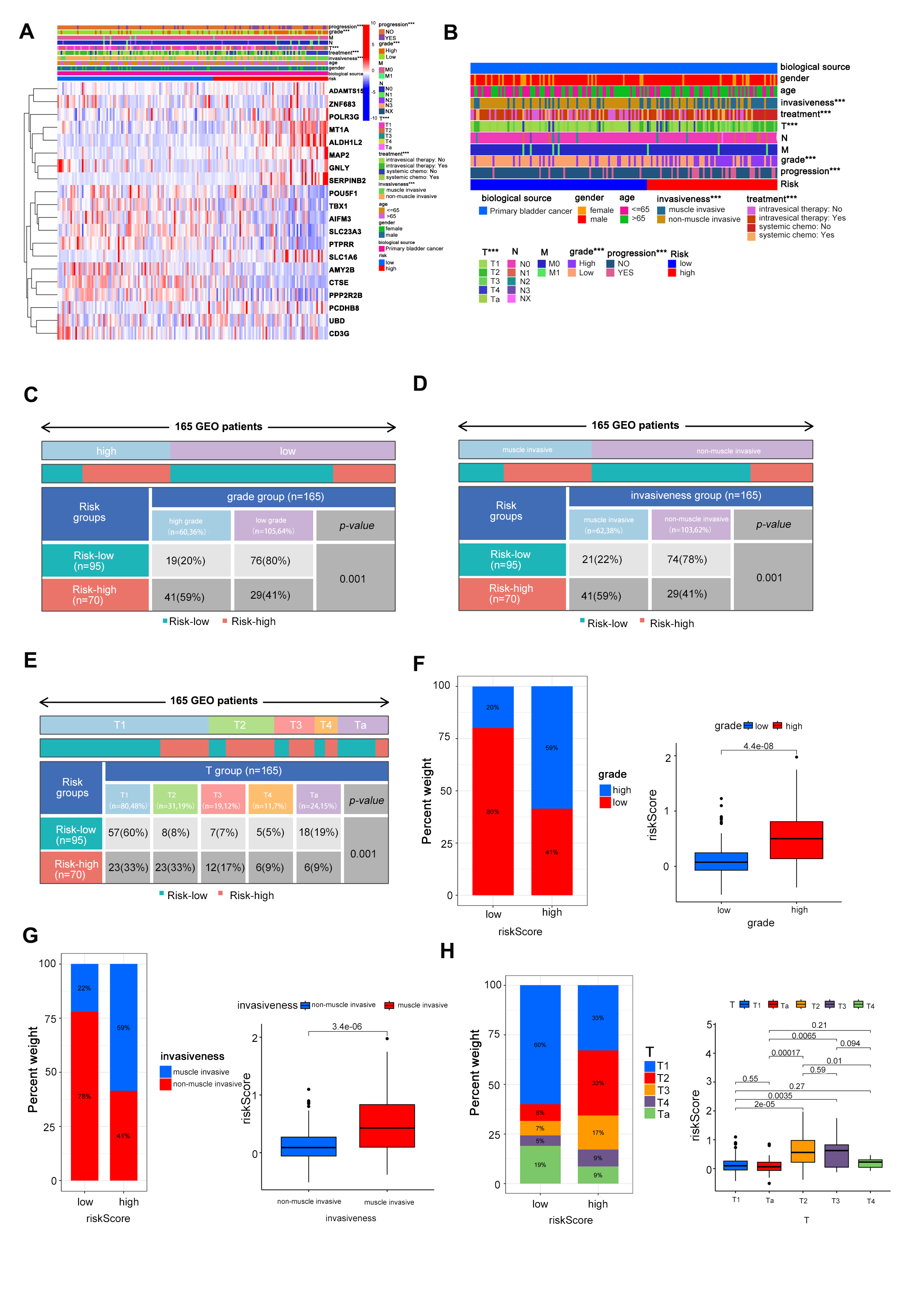

Supplement: Supplementary file 2 — Figure S2 [file JCMM-27-3995-s001.tif]

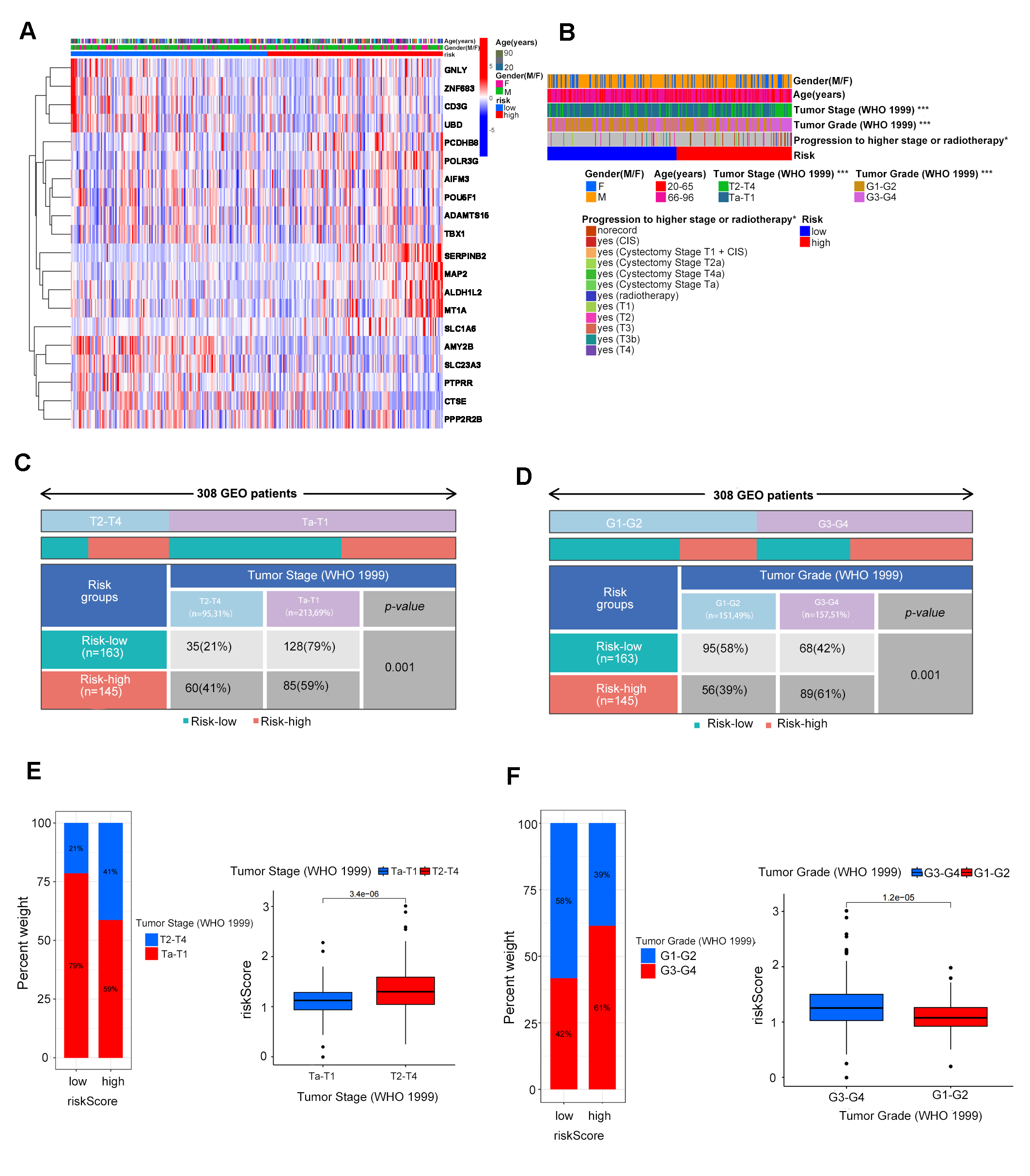

Supplement: Supplementary file 3 — Figure S3 [file JCMM-27-3995-s002.tiff]

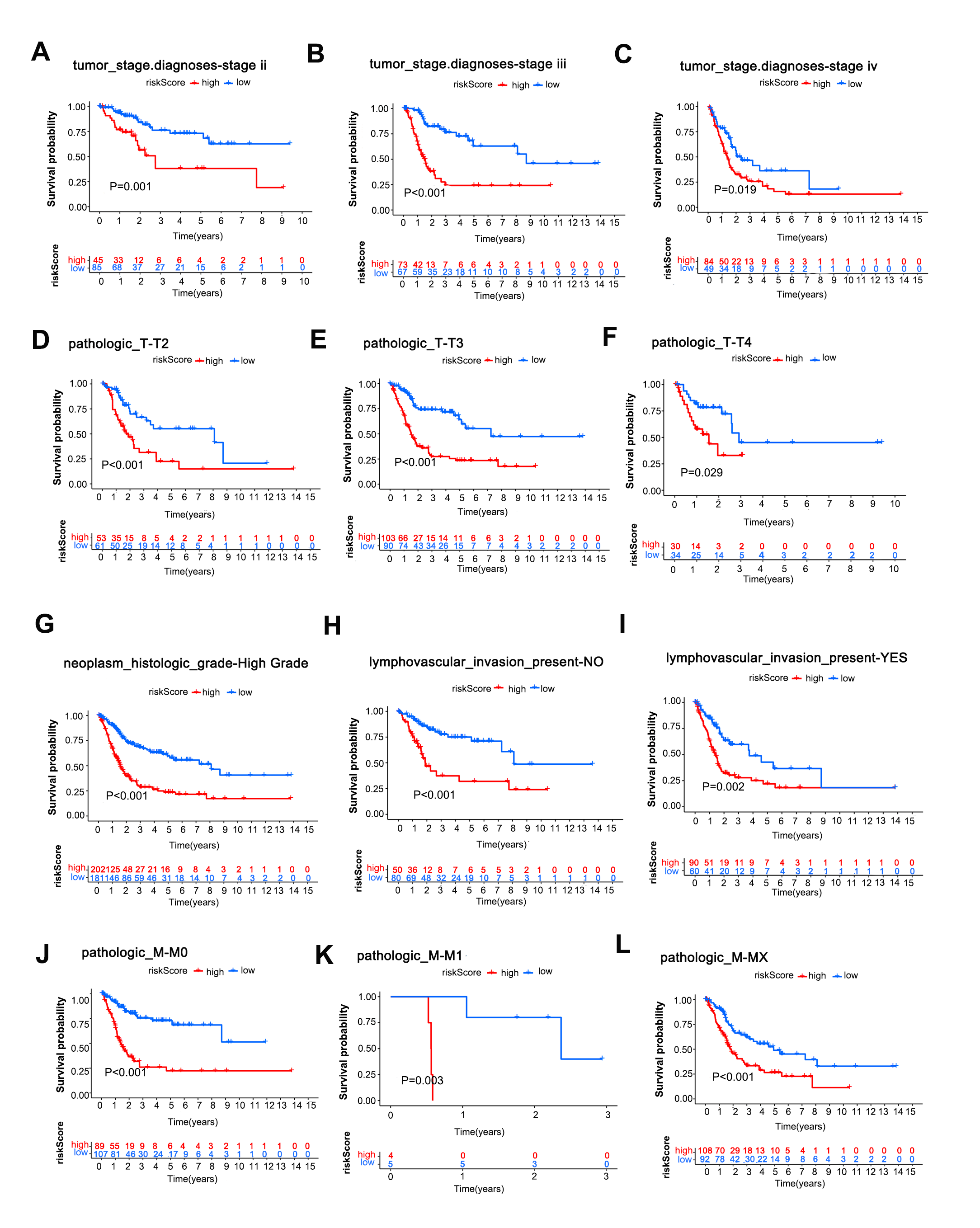

Supplement: Supplementary file 4 — Figure S4 [file JCMM-27-3995-s003.tif]
